# Real-Time Elastography of the Prostate

**DOI:** 10.1155/2014/180804

**Published:** 2014-05-22

**Authors:** D. Junker, T. De Zordo, M. Quentin, M. Ladurner, J. Bektic, W. Horniger, W. Jaschke, F. Aigner

**Affiliations:** ^1^Department of Radiology, Medical University Innsbruck, Anichstraße 35, 6020 Innsbruck, Austria; ^2^Department of Diagnostic and Interventional Radiology, Medical Faculty, Heinrich Heine University of Düsseldorf, Moorenstraße 5, 40225 Dusseldorf, Germany; ^3^Department of Urology, Medical University Innsbruck, Anichstraße 35, 6020 Innsbruck, Austria

## Abstract

Palpation of organs is one of the oldest clinical examination techniques, for instance, if you think of the palpation of the breast or the digital rectal examination of the prostate, where hard palpable regions are suspicious for cancer. This is the basic principle of real-time elastography, an ultrasound technique, which is able to visualise tissue elasticity. Since prostate cancer features an increased stiffness due to the higher cell and vessel density than the normal surrounding tissue, real-time elastography has been used for several years for prostate cancer detection. This review introduces the different techniques of ultrasound elastography and furthermore summarises its limitations and potentials.

## 1. Introduction 

Prostate cancer (PCa) is one of the most common cancers in men in the western world. The American Cancer Society estimates 241,740 new cases of PCa and 28,170 cases of death related to PCa in the USA for the year 2012 [1]. Elevated PSA serum values and/or positive findings in digital rectal examination (DRE) are suspicious for PCa. Histopathological evaluation of systematic biopsy (SB) cores is used to confirm or rule out cancer. In SB, the conventional transrectal ultrasound (TRUS) is primary utilised for biopsy guidance and not for cancer detection, because suspicious hypoechoic areas represent cancer in only 9–53% of cases in the grey-scale technique [2].

Despite the low specificity of PSA testing and the low sensitivity of SB, these techniques remain the standard of care for PCa diagnosis, mainly because of the high availability and low costs [3–5]. Nevertheless, on the one hand, this strategy misses significant PCa in a high percentage of patients and, on the other hand, detects many insignificant PCa, which leads to overdiagnosis and overtherapy [6].

Because of encouraging technical innovations and developments in prostate imaging, it is now possible to visualise PCa with high sensitivity [7, 8]. Beside PCa localisation, modern imaging modalities are capable of providing information about tumour volume, local staging, and cancer aggressiveness, which may be helpful for choosing the most appropriate therapy [8, 9].

Multiparametric MRI (mpMRI) and modern TRUS techniques are currently used for PCa detection [7, 10, 11]. In contrast to mpMRI and contrast-enhanced TRUS, real-time elastography (RTE) is a cheap and noninvasive tool, which can be performed by both radiologists and urologists.

## 2. Techniques

Ophir et al. described the principles of elastography in 1991 [12]. Krouskop et al. found a significant difference in stiffness between normal and neoplastic prostate and breast tissue [13]. To reduce the need for time-consuming calculations, Pesavento developed a fast cross-correlation technique. This technique became the basis for RTE under real-time conditions [14]. Currently, there are 2 different techniques which are used to demonstrate tissue elasticity of the prostate: strain elastography (SE) and shear-wave elastography (SWE).

### 2.1. Strain Elastography (SE)

SE is the most widely used technique to estimate tissue stiffness of the prostate. Elastograms are obtained by slight compression and decompression of the prostate, which is manually induced with the transrectal probe by the operator. To visualise the distribution of tissue elasticity, images are colour-coded, for example, from red (soft tissue) to blue (hard tissue). The elastography box should cover the entire gland and the surrounding tissues but avoid the bladder [15].

### 2.2. Shear-Wave Elastography (SWE)

In contrast to SE, SWE does not require a manually induced compression of the prostate. The tissue elasticity is calculated by measuring the velocity of shear-waves, which is different in soft and hard tissues of the prostate. Similar to SE, SWE can colour-code the distribution of tissue elasticity. Furthermore, this technique is able to generate an absolute value of tissue stiffness in kilopascal. The image can cover half of the gland in axial planes; thus, each side of the prostate should be examined separately [15, 16].

### 2.3. Quantification

**Figure 1:**
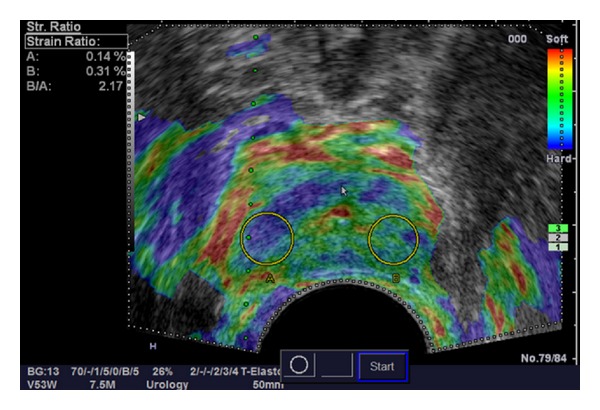
Obtaining a stiffness value by strain ratio measurement.

Obtaining a measurable value indicating the degree of tissue stiffness has been shown to be useful for differentiating between benign and malignant hard lesions, for example, in the breast [17]. To possibly reduce false positive findings of RTE of the prostate, Zhang et al. investigated the strain ratio index of SE, which is a semiquantitative measurement tool comparing the strain value of the target lesion with the background tissue [18] (Figure 1). They reported a significant difference of peak strain index values between benign and malignant lesions (*P* < 0.01). Furthermore, the cut-off of 17.44 peak strain index value yielded the highest sensitivity (74.5%) and specificity (83.3%) with an AUC of 0.90 for discriminating PCa.

In contrast to SE, SWE can provide absolute values in kilopascals. Regarding the negative predictive value, cut-off values of 35 and 37 kPa were reported for SWE, providing the best performance with a sensitivity, specificity, PPV, and NPV of 63%, 91%, 69.4%, and 91%, respectively [15, 16, 19].

### 2.4. Learning Curve

Heinzelbecker et al. assessed the learning curve of RTE of the prostate dividing the gland into 8 and 16 sectors [20]. They included 60 PCa patients before radical prostatectomy (RPE). All men were examined by a novice and an expert observer. Results of the novice observer were validated with those of the expert. The study group demonstrated valid results after 30 examinations for the 8-sector model when being trained by an expert. Regarding precision, more examinations enhanced the quality of the results as they could use the 16-sector model. Overall, they concluded that RTE of the prostate can be learned quickly by an unexperienced examiner and the examination itself is not very time-consuming.

However, the manual compression and decompression are relatively uncontrolled elements of SE with wide variability. Thus, images affected by artefacts have been found in up to 32% of cases [21]. Standardised compression using real-time balloon inflation has improved results, showing sensitivity and specificity up to 72.5% and 97.7%, respectively [22].

## 3. Physiology-Histology: Normal Patterns, False Positive, and False Negative Findings

### 3.1. Normal Patterns

**Figure 2:**
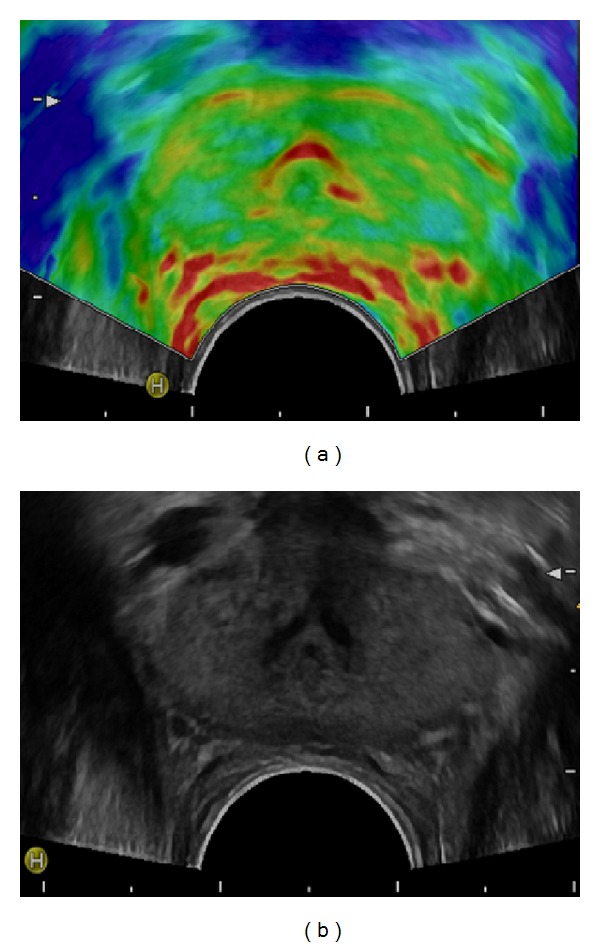
Elastogram shown on (a) demonstrates the normal sized prostate of homogeneous high elasticity (colour-coded green) in a healthy young patient; corresponding grey-scale image is shown on (b).

**Figure 3:**
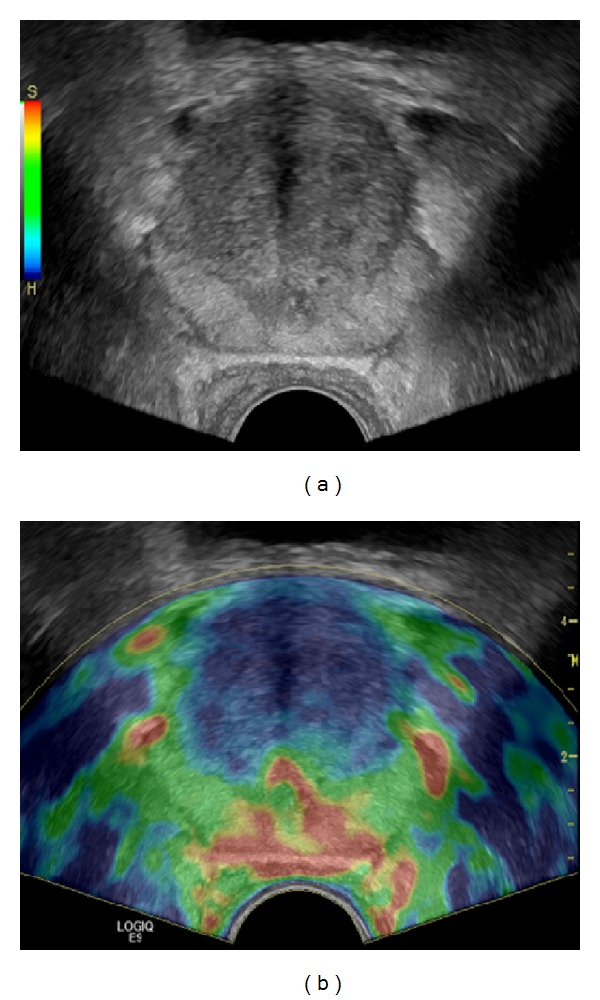
Elastogram shown on (b) demonstrates the enlarged prostate with benign prostatic hypertrophy of homogeneous high elasticity in the peripheral zone (colour-coded red to green) and of decreased elasticity in the inner gland (colour-coded blue) in an elderly patient; corresponding grey-scale image is shown on (a).

**Figure 4:**
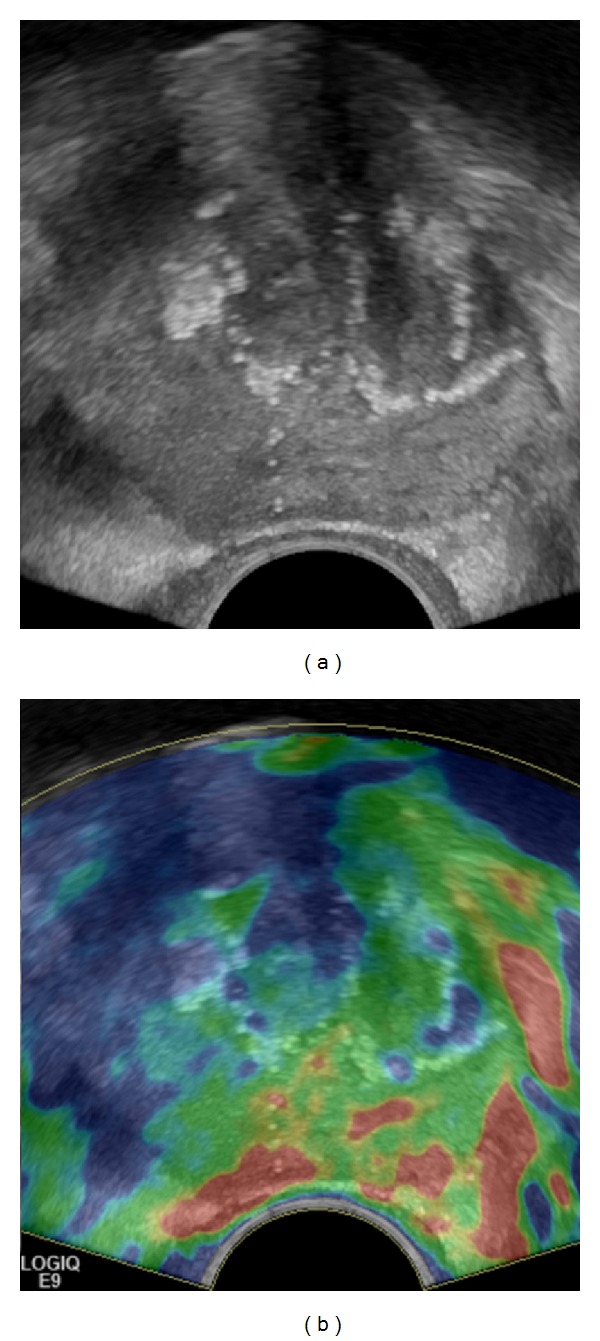
Elastogram shown on (b) demonstrates a disrupted soft rim sign in a patient with a huge tumour in the right gland suspicious of extracapsular extension; corresponding grey-scale image is shown on (a).

Goddi et al. selected 100 patients for a study of normal elastographic findings in prostates of various sizes [23]. To rule out pathologies, the study group included patients with no signs of inflammation, normal findings in the DRE, total PSA serum values <4 ng/mL, PSA ratio >0.18, PSA density < 0.04, no structural alterations in the peripheral zone (PZ) in the conventional TRUS, and prostate volumes between 20 cc and 100 cc. The normal PZ of the prostate was of intermediate elasticity (Figure 2), while the inner gland showed more heterogeneity. Furthermore, they demonstrated that elasticity patterns of the prostate which are investigated with SE depend on physiological changes, demonstrating an increasing stiffness with growing age and volume, especially in the inner gland (Figure 3). The absence of the soft rim artefact may indicate extracapsular extension (ECE) (Figure 4). These findings are in line with those of Correas et al., who reported for SWE that the entire prostate in young healthy patients is of homogeneous soft appearance and that the PZ remains soft and homogeneous, while the inner gland becomes heterogeneous, in the case of benign prostate hypertrophy (BPH) [24].

### 3.2. False Positive Findings

**Figure 5:**
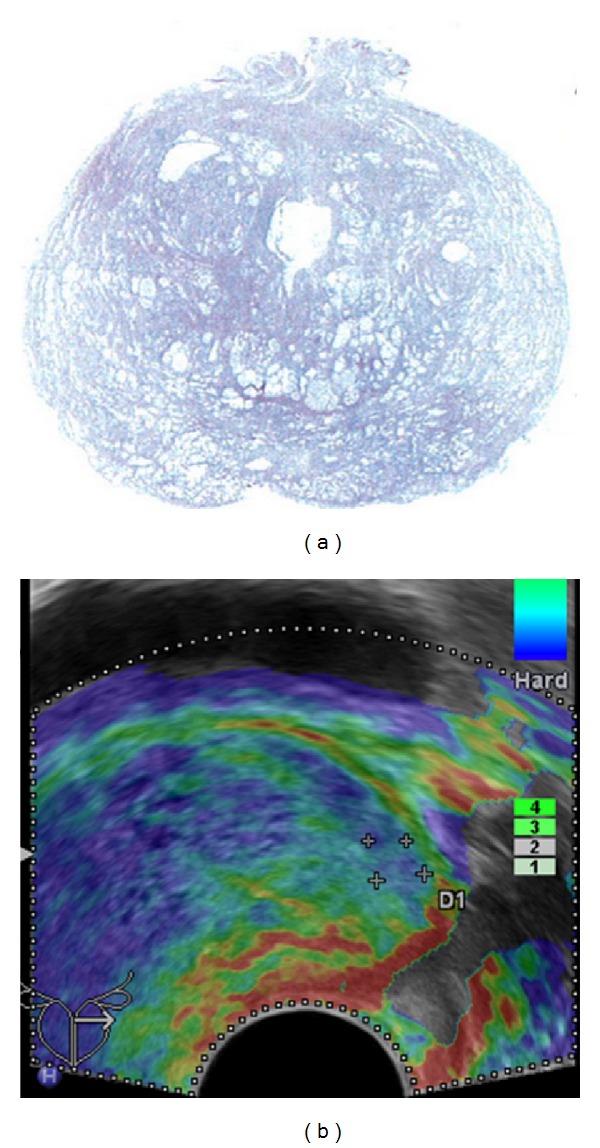
False positive finding: elastogram shown on (b) demonstrates a lesion of decreased elasticity in the left peripheral zone (colour-coded blue); corresponding whole-mount step section shown on (a) yielded an atrophic area.

Benign entities like prostatitis, fibrosis, atrophy, adenomyomatosis, and BPH may be associated with increased tissue stiffness and therefore may be difficult to distinguish from PCa. This can be responsible for low positive predictive values to the point of only 39%, as reported by our group when studying men with PSA serum values <4 ng/mL [25] (Figure 5).

### 3.3. False Negative Findings

**Figure 6:**
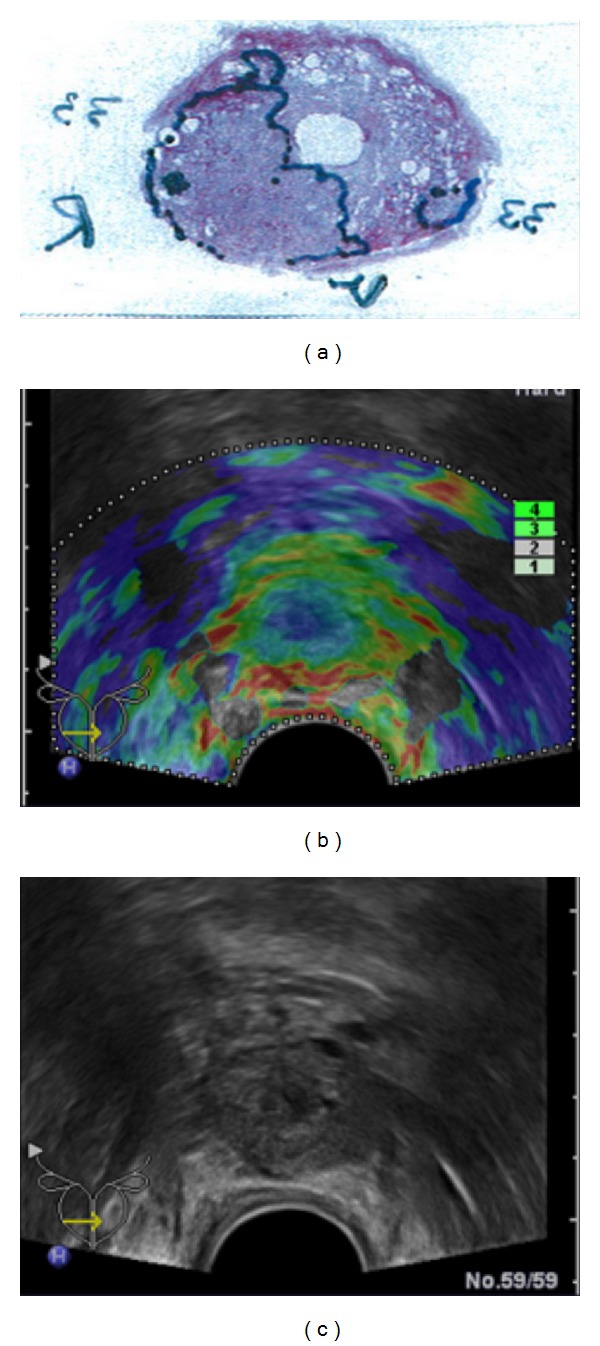
Elastogram shown on the left side demonstrates the dense cancer as a lesion of decreased elasticity in the right gland (colour-coded blue) but misses the sparse cancer in the left gland; corresponding whole-mount step section is shown above.

Langer et al. divided cancers into those with dense architecture on histology and into those with sparse architecture [26]. In particular, cancers with predominant Gleason pattern 3 (e.g., 3 + 3 or 3 + 4) may be of sparse architecture consisting of a mixture of normal tissue and cancerous tissue or possessing glands with dilated lumina and are therefore soft. For imaging, in general, this histological kind of tumour composition may be a problem. For diffusion-weighted MRI (DWI), Langer et al. demonstrated that all “invisible” tumours in DWI had predominant Gleason pattern 3 and showed sparse architecture on histology [26]. For RTE, we revealed similar results, since RTE visualises cancer due to the higher cell density compared to the normal surrounding tissue [13, 27]. Nearly all cancers missed on RTE in our study population with a significant tumour volume were also of sparse architecture and had predominant Gleason pattern 3. Nevertheless, sensitivity of PCa with a Gleason score ≥4 + 3 was very high, since a predominant Gleason pattern 4 or 5 indicates dense tumours [27] (Figure 6).

## 4. RTE Targeted Biopsy

**Figure 7:**
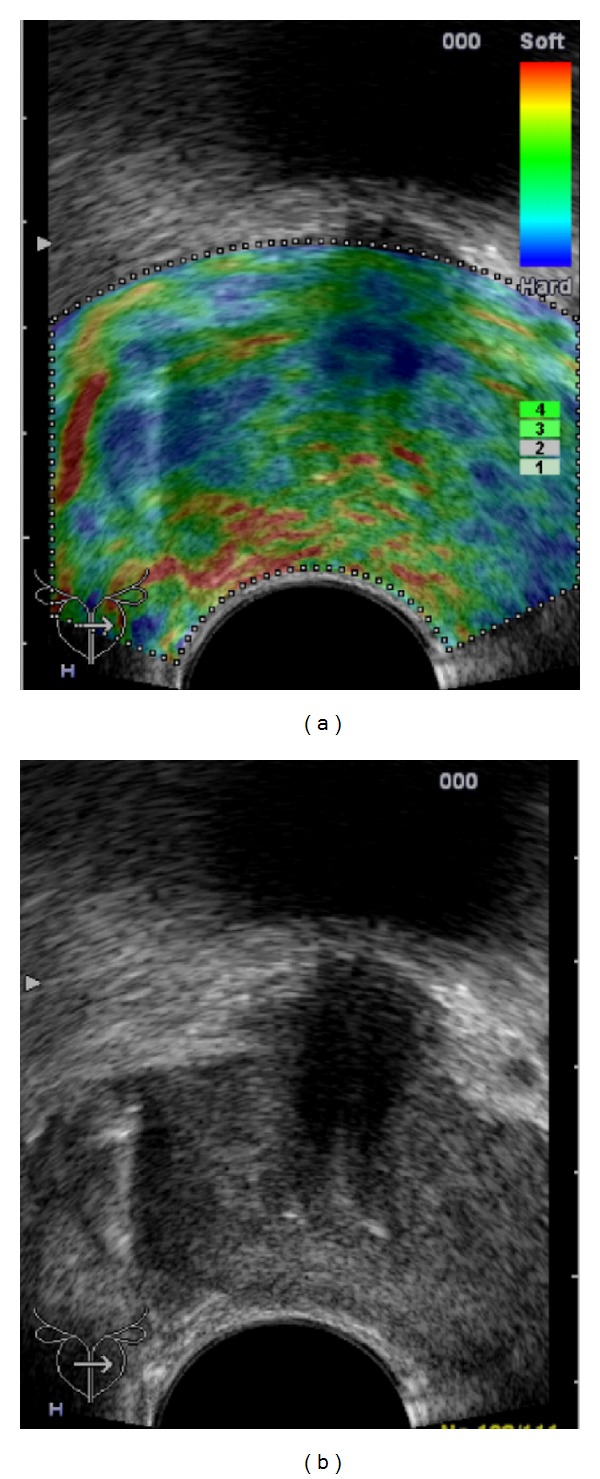
RTE targeted biopsy of a lesion with decreased elasticity (colour-coded blue) under real-time conditions is shown on the elastogram on (a); corresponding grey-scale image is shown on (b).

Many study groups have shown the usefulness of guiding the biopsy needle directly into lesions of the prostate which are suspicious for PCa on RTE (Figure 7).

In a series of 230 men, Pallwein et al. compared a 5-core RTE targeted biopsy with a 10-core SB and demonstrated a sensitivity of 84% for RTE. The PCa detection rate per patient was not significantly higher for the targeted approach compared to the systematic approach (30% versus 25%; McNemar's test: *P* = 0.134), but an RTE targeted core was 2.9-fold more likely to be cancer positive than a systematic core (McNemar's test: *P* < 0.001) [28].

Similar results were obtained in a study of Aigner et al., which included only patients with PSA serum levels <4 ng/mL [25]. Targeted biopsies with a maximum number of 5 cores were performed in PCa suspicious areas on RTE, followed by 10-core SB. The PCa detection rate per patient was nearly equal (21.3% versus 19.1%), whereas the cancer detection rate per core was 4.7-fold greater for targeted compared to systematic biopsy. Furthermore, a high number of patients were PCa positive (27 of 94; 28.7%), even in this low PSA group, which may suggest that RTE detects PCa independently of PSA serum values.

An interesting RTE targeted biopsy scheme was performed in a cohort of 178 men by Brock et al., who divided the prostate into sectors and limited the targeted approach to a 10-core biopsy to avoid additional cores and to reduce the overall number of biopsy cores [29]. If RTE did not visualise a suspicious area in a sector, the biopsy core was taken systematically; otherwise, an RTE targeted biopsy was performed. In their randomised prospective study, they compared the findings of these 178 patients with another study group of 175 men undergoing a 10-core conventional TRUS-guided biopsy. They showed a significantly higher PCa detection rate in the RTE group than in the control group (51.1% versus 39.4% (*P* = 0.027)). Overall sensitivity and specificity of PCa detection were 60.8% and 68.4%, respectively, for the RTE targeted approach.

A recently published study from Salomon et al. underlines the potential of RTE targeted biopsy [11]. They analysed 1024 patients who consecutively underwent a 4-core RTE targeted biopsy in addition to a randomised 10-core conventional TRUS-guided biopsy. PCa detection rate of the combined approach, of the 10-core SB, and of the 4-core RTE biopsy was 46.2% (*n* = 473), 39.1% (*n* = 400), and 29.0% (*n* = 297), respectively. RTE targeted biopsy also detected a further 72 patients with PCa who were not detected by systematic 10-core biopsy. Therefore, the additional use of RTE led to an incremental value of 18.3%. Of vital importance is the fact that 34 patients harboured significant PCa (primary Gleason pattern 4 or 5) diagnosed by RTE biopsy only.

Targeted biopsy under RTE guidance seems to have the advantage of the reliable detection of significant disease [30]. According to Nygård et al. the addition of RTE targeted cores to standard SB raised the negative predictive value from 79% to 97% for high-risk PCa. They concluded that a positive RTE is an independent marker of the detection of high-risk PCa, and a negative RTE argues against such a diagnosis. Nevertheless, both Salomon et al. and Nygård et al. did not recommend RTE targeted biopsy alone, since this approach missed a high proportion of patients with PCa in their studies [11, 30].

Despite these promising results, there are heterogeneous results in the literature for the general benefit of RTE targeted biopsy for PCa diagnosis. For instance, Taverna et al. found a relatively low sensitivity, specificity, positive predictive value (PPV), and negative predictive value (NPV) of 24.4%, 65.7%, 21.9%, and 68.6%, respectively, in 102 men with PSA serum values ranging from 2.5 ng/mL to 10 ng/mL for RTE. They concluded that RTE cannot be recommended for standard PCa diagnosis [31]. Therefore, prospective multicentre studies would be desirable to show the true value of RTE targeted biopsy. A first big multicentre study was already initiated by 10 European centres (ESUI 12/01 HI-RTE); the first results are expected in the coming months.

Nevertheless, nearly all studies have shown that the RTE targeted approach detects high-risk PCa more reliably than SB, requires a reduced number of cores for PCa detection, and enhances the overall sensitivity in the combined biopsy setting.

## 5. RTE before Radical Prostatectomy (RPE)

Since SB may miss PCa, it serves as an incomplete gold standard to calculate the overall diagnostic accuracy for other imaging modalities [32]. A comparison with RPE specimens allows for the exact localisation of PCa, but the investigator might be biased by his knowledge of the patient's disease.

### 5.1. PCa Localization

Studies comparing preoperative elastograms with whole-mount step sections after RPE showed a sensitivity of 50–87%, a specificity of 72–92% for correct PCa localisation, and an overall PPV of 67–88% and a NPV of 44–83% [33–36]. Nygård et al. demonstrated the benefit of adding new biomarkers, like PCA-3, to RTE findings for the detection of significant disease [37].

### 5.2. PCa Index Lesion

One of the key requirements of imaging is to visualise the PCa index lesion, since this may determine the clinical prognosis [38]. Walz et al. and Brock et al. considered the index lesion as the largest lesion suspicious for PCa [38, 39]. Walz et al. revealed a sensitivity, specificity, negative predictive value, positive predictive value, and diagnostic accuracy of 58.8%, 43.3%, 54.1%, 48.1%, and 51.6% for RTE alone, but combining biopsy data and RTE findings increased the values to 84.9%, 48.4%, 61.9%, 75.0%, and 66.1%, respectively [38]. Brock et al. achieved a positive predictive value of 65.1% for the index lesion with RTE alone, but combining contrast-enhanced TRUS and RTE findings increased the value to 89.7% [39].

### 5.3. Tumour Size and Tumour Volume

Our study group investigated PCa detection rates for RTE with regard to tumour size and tumour volume in a whole-mount step section analysis [27]. Concerning the tumour size, RTE detected 6 out of 62 cancer lesions with a maximum diameter of 0–5 mm (9.7%), 10 out of 37 with a maximum diameter of 6–10 mm (27%), 24 out of 34 with a maximum diameter of 11–20 mm (70.6%), and 14 out of 14 with a maximum diameter of >20 mm (100%). Concerning the tumour volume, RTE detected 40 out of 48 cancer lesions with a tumour volume ≥0.2 cm^3^ (83.3%) and 31 out of 34 with a tumour volume ≥0.5 cm^3^ (91.2%). Roethke et al. found similar results for endorectal MRI; therefore, we may conclude that imaging of PCa is generally influenced by tumour size and tumour volume [40].

### 5.4. Local Staging

For the prediction of ECE, Brock et al. showed a sensitivity of 38% and a specificity of 96% for RTE (Figure 6). This represents an improvement in comparison to the grey-scale TRUS but still remains inadequate for clinical practice [35]. In contrast, Pelzer et al. found a sensitivity of 79% and a specificity of 89% for the detection of ECE by RTE [41]. Zhu et al. showed a sensitivity for PCa detection confined to the capsule of 51.6% and of 79.3% with ECE and stated that this might be related to the fact that tumours with larger diameters, which were more likely to be detected by RTE, were more likely to extend throughout the capsule [42].

## 6. RTE in Comparison to Multiparametric MRI (mpMRI)

**Figure 8:**
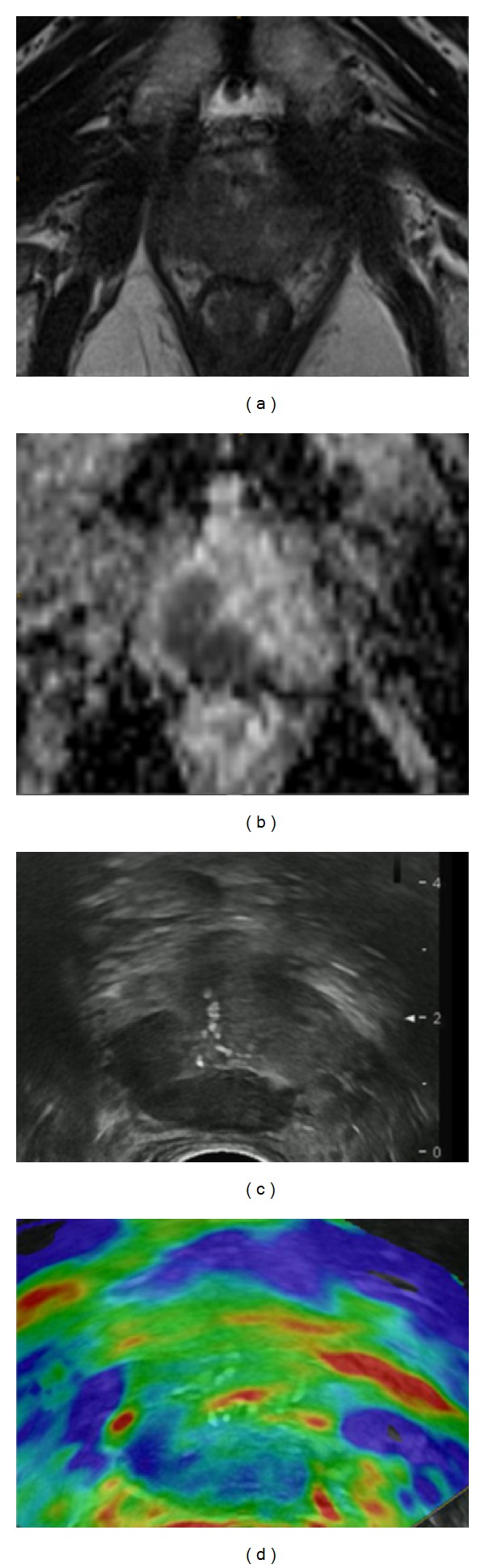
PCa (dorsal right peripheral zone) is shown on both modalities. (a) T2-weighted imaging showing the cancer as an area of decreased signal intensity; (b) apparent diffusion coefficient showing the cancer as an area of restricted diffusion; (c) grey-scale image showing the cancer as an area of decreased echogenicity; (d) elastogram showing the cancer as an area of decreased elasticity (colour-coded blue).

It is of great interest to compare RTE with MRI for PCa diagnosis, since MRI is a very promising imaging technique for the prostate and is also considered a gold standard (Figure 8). There are only 4 studies which have dealt with this issue [41, 43–45].

### 6.1. Detection Rates and Localisation

In a small series, Aigner et al. investigated the potential of RTE in comparison with T2-weighted endorectal MRI (T2w-MRI) for PCa detection using a biopsy specimen as the reference standard and showed similar results [43]. RTE showed sensitivity rates and negative predictive values per patient of 84.6% and 86.7%, respectively, a T2w-MRI of 84.6% and 83.3% for MRI.

Sumura et al. used whole-mount step sections as the reference standard and reported a superiority of RTE detection rates to those of MRI (74.1% versus 47.4%) and nearly equal detection rates for RTE alone on both the anterior side (75.0%) and the posterior side (73.7%) of the prostate [44]. Furthermore, a higher tumour detection rate for RTE was observed for tumours with a higher Gleason score and larger tumour volume.

While the study conducted by Sumura et al. used T2w-MRI and contrast-enhanced MRI (ceMRI) only, Pelzer et al. used a multiparametric approach including T2w-MRI, DWI, and ceMRI following the recommendations from the European Society of Urogenital Radiology (ESUR) [41, 46]. Summarising their results, RTE showed advantages in apical and middle parts of the prostate for PCa detection, whereas mpMRI provided advantages in the glands' base and transitional zone (TZ). Both RTE and mpMRI had limitations, particularly in basal and ventral parts. Furthermore, most of the undetected tumours were of low tumour volume and Gleason score.

These data are in line with a study by Junker et al., who also compared RTE findings with those of mpMRI in a whole-mount step section analysis [45]. Histopathology revealed 61 cancer lesions ≥0.2 cm³. RTE detected 78% cancer lesions in the PZ and 18.2% in the TZ, while mpMRI detected 90% and 72.2%, respectively. Significant differences between both modalities were found for the TZ and anterior parts in prostates with a volume >40 mL (*P* < 0.05). Detection rates for high-risk PCa (Gleason score ≥4 + 3) and cancer lesions with volumes > 0.5 cm³ were 93.8% and 80.5%, respectively, for RTE, and 87.5% and 92.7%, respectively, for mpMRI. The authors concluded that RTE and mpMRI detected high-risk PCa with high sensitivity, but mpMRI seems to have advantages in tumour volume assessment and in the detection of PCa in the TZ and in anterior-localised PCa within prostate glands with volumes of >40 mL.

### 6.2. Considerations for Focal Therapy

**Figure 9:**
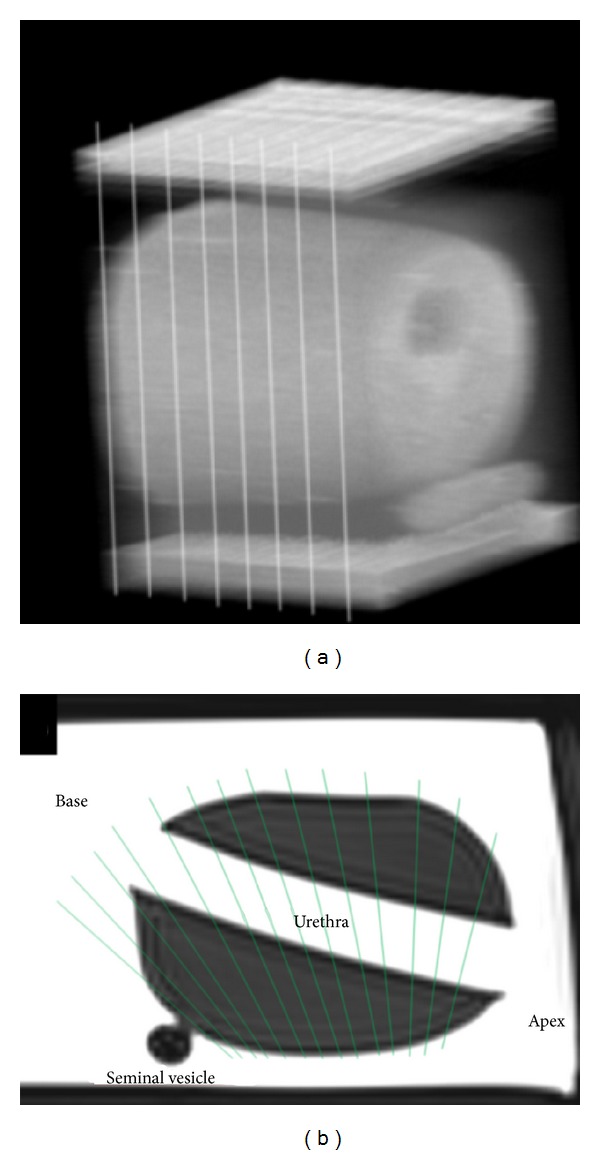
Whole-mount step sections and MRI obtain planes with nearly 90° to the urethra shown in image (a); RTE using an endfire probe obtains planes with different angles in different prostate sectors shown in image (b).

**Figure 10:**
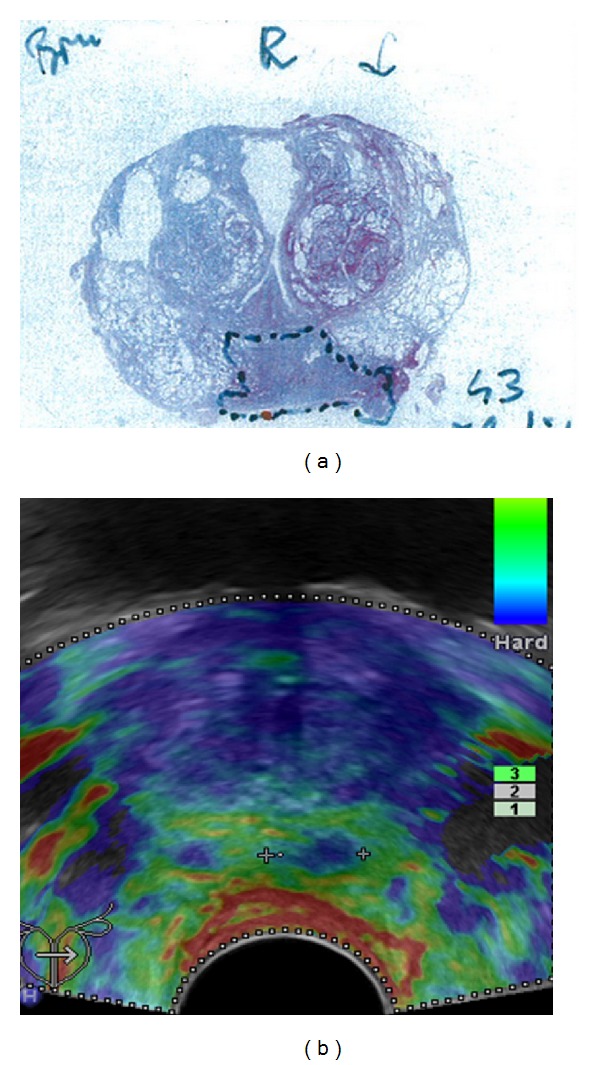
Tumour volume demonstration of a Gleason score 7 PCa on the whole-mount step section (a) and on RTE (b) RTE demonstrates the cancer on the base of the prostate smaller than the corresponding whole-mount step section.

Curiel et al. assessed the volume of HIFU lesions in prostate glands and found that elastography generally underestimated tumour volume in comparison with MRI [47]. These findings are in agreement with our observations: Spearman's rank correlation was 0.72 for mpMRI and only 0.46 for RTE when comparing volume measurements of cancer lesions with histopathology [45]. The explanation might be the usage of an endfire probe for RTE in our population, which scans the prostate at different angles compared to the whole-mount step sections or to mpMRI (Figures 9 and 10). When additionally keeping in mind the results for detecting the index lesion and for low-risk PCa, which may be candidates for focal therapy, it seems that RTE alone does not have the potential for this approach [11, 38, 39, 48].

## 7. Summary

Currently, PCa diagnosis is in a paradigm shift. On the one hand, imaging was and is still used to improve the PCa detection rate. On the other hand, some urologists have more recently indicated the desire to detect clinically significant cancers only in order to prevent overdiagnosis and overtreatment, which is also associated with higher costs and complications. RTE may be particularly suitable for this purpose, since RTE is of limited value in the detection of small cancers and may miss carcinomas with a primary Gleason pattern of 3. In the end, it is up to the clinician to decide which approach is to be favoured.

**Figure 11:**
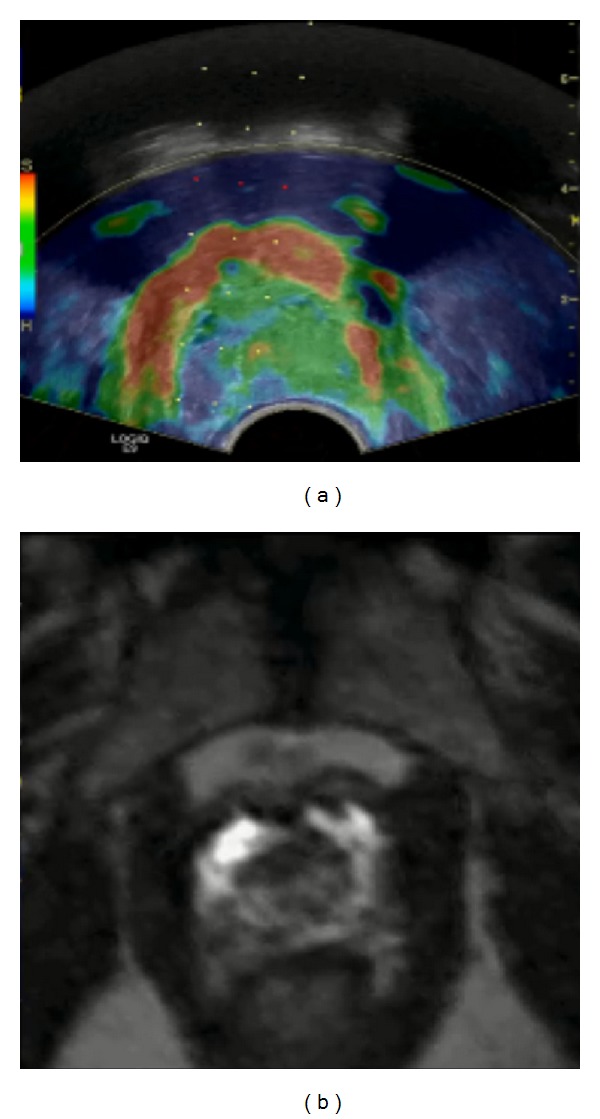
RTE demonstrates the low signal intensity nodule on T2-weighted image (b) as a lesion of decreased elasticity ((a), colour-coded blue).

**Figure 12:**
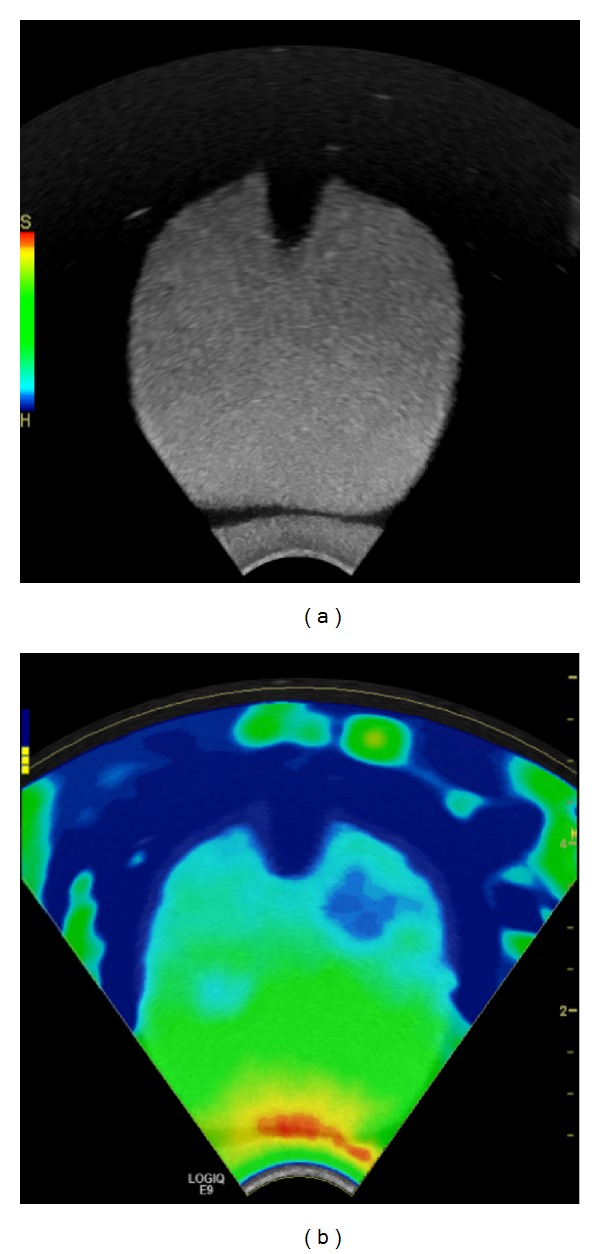
Anterior lesion in a prostate phantom not visible on grey-scale ultrasound (a), but visible on elastogram ((b), colour-coded blue).

Therefore, we do not summarise advantages and disadvantages of RTE but report the characteristics only:

- it can be performed by both urologists and radiologists,
- biopsy can be performed under real-time conditions,
- it raises the overall sensitivity for biopsy combined with SB,
- it is independent of PSA serum values,
- it detects cancer of significant volume and significant disease with high sensitivity,
- it can characterise findings on other imaging modalities (Figure 11),
- it can demonstrate PCa not visible on other imaging modalities (Figure 12),
- it may have problems detecting PCa in the TZ and anterior-localised PCa within big-sized prostates,
- it may have problems diagnosing PCa with sparse architecture,
- it has a considerable number of false positive findings,
- it has low evidence level due to the lack of multicentre studies.

**Figure 13:**
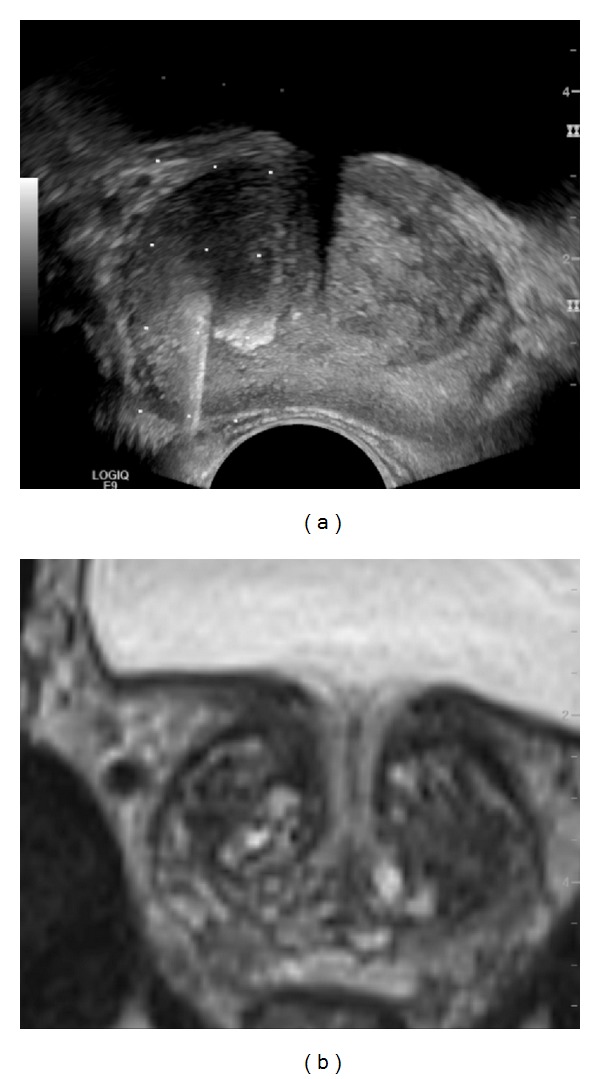
Ultrasound/mpMRI image fusion guided biopsy of the right peripheral zone of the prostate: low signal intensity nodule on T2-weighted image (b) directly biopsied under live-ultrasound guidance (a).

The mentioned characteristics have led to the following protocol for PCa diagnosis at our institution: in the primary biopsy setting we combine RTE targeted and systematic biopsy. RTE targeted cores are taken only in case of cancer-suspicious lesions. In the case of a prior negative biopsy with the combined approach but ongoing cancer suspicion, we investigate the prostate with mpMRI at 3T. If there are cancer-suspicious lesions on mpMRI, patients undergo an ultrasound/mpMRI image fusion guided biopsy (Figure 13).

## References

[1] Siegel R, Naishadham D, Jemal A (2012). Cancer statistics, 2012. *CA Cancer Journal for Clinicians*.

[2] Dähnert WF, Hamper UM, Eggleston JC (1986). Prostatic evaluation by transrectal sonography with histopathologic correlation: the echopenic appearance of early carcinoma. *Radiology*.

[3] Gómez Veiga F, Ponce Reixa J, Barbagelata López A, Fernández Rosado E, González Martín M (2006). Current role of PSA and other markers in the diagnosis of prostate cancer. *Archivos Espanoles de Urologia*.

[4] Norberg M, Egevad L, Holmberg L, Sparén P, Norlén BJ, Busch C (1997). The sextant protocol for ultrasound-guided core biopsies of the prostate underestimates the presence of cancer. *Urology*.

[5] Heidenreich A, Bellmunt J, Bolla M (2011). EAU guidelines on prostate cancer. Part I: screening, diagnosis, and treatment of clinically localised disease. *Actas Urologicas Espanolas*.

[6] Borza T, Konijeti R, Kibel AS (2013). Early detection, PSA screening, and management of over-diagnosis. *Hematology/Oncology Clinics of North America*.

[7] Kurhanewicz J, Vigneron D, Carroll P, Coakley F (2008). Multiparametric magnetic resonance imaging in prostate cancer: present and future. *Current Opinion in Urology*.

[8] Aigner F, Schäfer G, Steiner E (2012). Value of enhanced transrectal ultrasound targeted biopsy for prostate cancer diagnosis: a retrospective data analysis. *World Journal of Urology*.

[9] Panebianco V, Sciarra A, Marcantonio A (2012). Conventional imaging and multiparametric magnetic resonance (MRI, MRS, DWI, MRP) in the diagnosis of prostate cancer. *Journal on Nuclear Medicine and Molecular Imaging*.

[10] Zhao HX, Xia CX, Yin HX, Guo N, Zhu Q (2013). The value and limitations of contrast-enhanced transrectal ultrasonography for the detection of prostate cancer. *European Journal of Radiology*.

[11] Salomon G, Drews N, Autier P (2014). Incremental detection rate of prostate cancer by HI-real time elastography targeted biopsies in combination with a conventional 10 core biopsy in 1024 consecutive men. *BJU International*.

[12] Ophir J, Cespedes I, Ponnekanti H, Yazdi Y, Li X (1991). Elastography: a quantitative method for imaging the elasticity of biological tissues. *Ultrasonic Imaging*.

[13] Krouskop TA, Wheeler TM, Kallel F, Garra BS, Hall T (1998). Elastic moduli of breast and prostate tissues under compression. *Ultrasonic Imaging*.

[14] Pesavento A (1999). A time-efficient and accurate strain estimation concept for ultrasonic elastography using iterative phase zero estimation. *IEEE Transactions on Ultrasonics, Ferroelectrics, and Frequency Control*.

[15] Cosgrove D, Piscaglia F, Bamber J (2013). EFSUMB guidelines and recommendations on the clinical use of ultrasound elastography. Part 2: clinical applications. *Ultraschall in der Medizin*.

[16] Barr RG, Memo R, Schaub CR (2012). Shear wave ultrasound elastography of the prostate: initial results. *Ultrasound Quarterly*.

[17] Cho N, Moon WK, Kim HY, Chang JM, Park SH, Lyou CY (2010). Sonoelastographic strain index for differentiation of benign and malignant nonpalpable breast masses. *Journal of Ultrasound in Medicine*.

[18] Zhang Y, Tang J, Li YM (2012). Differentiation of prostate cancer from benign lesions using strain index of transrectal real-time tissue elastography. *European Journal of Radiology*.

[19] Correas JM, Khairoune A, Tissier AM, Vassiliu V, Eiss D, Hélénon O (2012). *Trans-Rectal Quantitative Shear Wave Elastography: Application to Prostate Cancer. A Feasibility Study. Abstract*.

[20] Heinzelbecker J, Weiss C, Pelzer AE (2014). A learning curve assessment of real-time sonoelastography of the prostate. *World Journal of Urology*.

[21] Tsutsumi M, Miyagawa T, Matsumura T (2010). Real-time balloon inflation elastography for prostate cancer detection and initial evaluation of clinicopathologic analysis. *American Journal of Roentgenology*.

[22] Sumura MY, Naoko A, Takeo H, Koji W, Satoshi H (2010). Real-time balloon inflation elastography of prostate might surpass MRI for detection of prostate cancer. *Journal of Urology*.

[23] Goddi A, Sacchi A, Magistretti G, Almolla J (2011). Transrectal real-time elastography of the prostate: normal patterns. *Journal of Ultrasound*.

[24] Correas JM, Drakonakis E, Isidori AM (2013). Update on ultrasound elastography: miscellanea. Prostate, testicle, musculoskeletal. *European Journal of Radiology*.

[25] Aigner F, Pallwein L, Junker D (2010). Value of real-time elastography targeted biopsy for prostate cancer detection in men with prostate specific antigen 1.25 ng/ml or greater and 4.00 ng/ml or less. *Journal of Urology*.

[26] Langer DL, Van Der Kwast TH, Evans AJ (2008). Intermixed normal tissue within prostate cancer: effect on MR imaging measurements of apparent diffusion coefficient and T2-sparse versus dense cancers. *Radiology*.

[27] Junker D, Schäfer G, Aigner F (2012). Potentials and limitations of real-time elastography for prostate cancer detection: a whole-mount step section analysis. *The Scientific World Journal*.

[28] Pallwein L, Mitterberger M, Struve P (2007). Comparison of sonoelastography guided biopsy with systematic biopsy: impact on prostate cancer detection. *European Radiology*.

[29] Brock M, von Bodman C, Palisaar RJ (2012). The impact of real-time elastography guiding a systematic prostate biopsy to improve cancer detection rate: a prospective study of 353 patients. *The Journal of Urology*.

[30] Nygård Y, Haukaas SA, Halvorsen OJ (2014). A positive real-time elastography is an independent marker for detection of high-risk prostate cancers in the primary biopsy setting. *BJU International*.

[31] Taverna G, Magnoni P, Giusti G (2013). Impact of real-time elastography versus systematic prostate biopsy method on cancer detection rate in men with a serum prostate-specific antigen between 2.5 and 10 ng/mL. *ISRN Oncology*.

[32] Djavan B, Ravery V, Zlotta A (2001). Prospective evaluation of prostate cancer detected on biopsies 1, 2, 3 and 4: when should we stop?. *Journal of Urology*.

[33] Brock M, von Bodman C, Sommerer F (2011). Comparison of real-time elastography with grey-scale ultrasonography for detection of organ-confined prostate cancer and extra capsular extension: a prospective analysis using whole mount sections after radical prostatectomy. *BJU International*.

[34] Pallwein L, Mitterberger M, Struve P (2007). Real-time elastography for detecting prostate cancer: preliminary experience. *BJU International*.

[35] Salomon G, Köllerman J, Thederan I (2008). Evaluation of prostate cancer detection with ultrasound real-time elastography: a comparison with step section pathological analysis after radical prostatectomy. *European Urology*.

[36] Walz J, Marcy M, Maubon T (2011). Real time elastography in the diagnosis of prostate cancer: comparison of preoperative imaging and histology after radical prostatectomy. *Progres en Urologie*.

[37] Nygård Y, Haukaas SA, Waage JE (2013). Combination of real-time elastography and urine prostate cancer gene 3 (PCA3) detects more than 97% of significant prostate cancers. *Scandinavian Journal of Urology*.

[38] Walz J, Marcy M, Pianna JT (2011). Identification of the prostate cancer index lesion by real-time elastography: considerations for focal therapy of prostate cancer. *World Journal of Urology*.

[39] Brock M, Eggert T, Palisaar RJ (2013). Multiparametric ultrasound of the prostate: adding contrast enhanced ultrasound to real-time elastography to detect histopathologically confirmed cancer. *The Journal of Urology*.

[40] Roethke MC, Lichy MP, Jurgschat L (2011). Tumorsize dependent detection rate of endorectal MRI of prostate cancer—a histopathologic correlation with whole-mount sections in 70 patients with prostate cancer. *European Journal of Radiology*.

[41] Pelzer AE, Heinzelbecker J, Weiß C (2013). Real-time sonoelastography compared to magnetic resonance imaging using four different modalities at 3.0 T in the detection of prostate cancer: strength and weaknesses. *European Journal of Radiology*.

[42] Zhu Y, Chen Y, Qi T (2014). Prostate cancer detection with real-time elastography using a bi-plane transducer: comparison with step section radical prostatectomy pathology. *World Journal of Urology*.

[43] Aigner F, Pallwein L, Schocke M (2011). Comparison of real-time sonoelastography with T2-weighted endorectal magnetic resonance imaging for prostate cancer detection. *Journal of Ultrasound in Medicine*.

[44] Sumura M, Shigeno K, Hyuga T, Yoneda T, Shiina H, Igawa M (2007). Initial evaluation of prostate cancer with real-time elastography based on step-section pathologic analysis after radical prostatectomy: a preliminary study. *International Journal of Urology*.

[45] Junker D, Schäfer G, Kobel C (2014). Comparison of real-time elastography and multiparametric MRI for prostate cancer detection: a whole-mount step-section analysis. *American Journal of Roentgenology*.

[46] Barentsz JO, Richenberg J, Clements R (2012). European society of urogenital radiology. ESUR prostate MR guidelines. *European Radiology*.

[47] Curiel L, Souchon R, Rouvière O, Gelet A, Chapelon JY (2005). Elastography for the follow-up of high-intensity focused ultrasound prostate cancer treatment: initial comparison with MRI. *Ultrasound in Medicine and Biology*.

[48] Smeenge M, Barentsz J, Cosgrove D (2012). Role of transrectal ultrasonography (TRUS) in focal therapy of prostate cancer: report from a Consensus Panel. *BJU International*.

